# Adhesion, Growth, and Maturation of Vascular Smooth Muscle Cells on Low-Density Polyethylene Grafted with Bioactive Substances

**DOI:** 10.1155/2013/371430

**Published:** 2013-03-24

**Authors:** Martin Parizek, Nikola Slepickova Kasalkova, Lucie Bacakova, Zdenek Svindrych, Petr Slepicka, Marketa Bacakova, Vera Lisa, Vaclav Svorcik

**Affiliations:** ^1^Institute of Physiology, Academy of Sciences of the Czech Republic, Videnska 1083, 142 20 Prague 4-Krc, Czech Republic; ^2^Institute of Chemical Technology, Technicka 5, 166 28 Prague 6-Dejvice, Czech Republic

## Abstract

The attractiveness of synthetic polymers for cell colonization can be affected by physical, chemical, and biological modification of the polymer surface. In this study, low-density polyethylene (LDPE) was treated by an Ar^+^ plasma discharge and then grafted with biologically active substances, namely, glycine (Gly), polyethylene glycol (PEG), bovine serum albumin (BSA), colloidal carbon particles (C), or BSA+C. All modifications increased the oxygen content, the wettability, and the surface free energy of the materials compared to the pristine LDPE, but these changes were most pronounced in LDPE with Gly or PEG, where all the three values were higher than in the only plasma-treated samples. When seeded with vascular smooth muscle cells (VSMCs), the Gly- or PEG-grafted samples increased mainly the spreading and concentration of focal adhesion proteins talin and vinculin in these cells. LDPE grafted with BSA or BSA+C showed a similar oxygen content and similar wettability, as the samples only treated with plasma, but the nano- and submicron-scale irregularities on their surface were more pronounced and of a different shape. These samples promoted predominantly the growth, the formation of a confluent layer, and phenotypic maturation of VSMC, demonstrated by higher concentrations of contractile proteins alpha-actin and SM1 and SM2 myosins. Thus, the behavior of VSMC on LDPE can be regulated by the type of bioactive substances that are grafted.

## 1. Introduction

Construction of tissue replacements and tissue engineering are very important areas of contemporary medicine and biotechnology. They have great potential for the future, due to increased life expectancy, civilization disorders, and thus increased requirements for medical care. Advanced tissue replacements consist of two basic components: cells and cell carriers. Artificial materials are usually applied as cell carriers, and for this purpose they should be adapted to act as analogues of the extracellular matrix, that is, to control the adhesion, growth, phenotypic maturation, and proper functioning of the cells. Synthetic polymers are an important type of materials that can be used for constructing substitutes for various tissues of the human body. These materials have a wide range of advantages, such as relatively easy availability and low cost, defined and versatile chemical composition, tunable mechanical properties, and tailored biodegradability at physiological conditions. These properties have made these polymers an obvious choice of material for many biotechnological and medical applications, for example, as growth supports for cell cultures *in vitro* or for constructing nonresorbable, fully resorbable, or semiresorbable vascular prostheses [1–4], artificial heart valves [5], bone and joint replacements [6, 7], implants for plastic surgery [8], bioartificial skin [9], and carriers for cell, drug or gene delivery [10]; for a review, see [11–14]. 

For biomedical applications, it is generally accepted that synthetic polymeric materials have to be biocompatible; that is, they must match the mechanical properties of the replaced tissue and not act as cytotoxic, mutagenic, or immunogenic. In addition, the physicochemical characteristics of the surface of these biomaterials are of great importance, because they can directly influence and control the cell adhesion, spreading, and signaling events that further regulate a wide range of biological functions, for example, cell growth, differentiation, and extracellular matrix synthesis [15]. 

However, in their pristine state, many polymeric materials have unfavorable physical and chemical surface properties, which are limiting for their colonization with cells and for their integration with the surrounding tissues in the patient's organism. A typical example is the high hydrophobicity of synthetic polymers; that is, the water drop contact angle on the material surface is often higher than 90°. Fortunately, a wide range of physical and chemical modifications is available that can be used to create more hydrophilic bioactive surfaces attractive for cell colonization. For example, the polymers can be irradiated with ions [2, 3], with ultraviolet light [14, 16, 17], or exposed to plasma [18]. These treatments induce degradation of the polymer chains, release of noncarbon atoms, and creation of radicals. These radicals react with oxygen in the ambient atmosphere, leading to the formation of oxygen-containing functional chemical groups on the polymer surface (i.e., carbonyl, carboxyl, hydroxyl, ether, or ester groups). These groups enhance the polymer polarity and wettability and promote the adsorption of cell adhesion-mediating molecules in appropriate geometrical conformations, which enable specific amino acid sequences (e.g., RGD) in these molecules to be reached by cell adhesion receptors. In addition, conjugated double bonds between carbon atoms are created, and this renders the polymer surface electrically conductive. It is known that the electrical conductivity of a material surface enhances its attractiveness for cell colonization, even without active electrical stimulation (for a review, see [11–13, 19]). 

In addition, the radicals, oxygen-containing groups, and double bonds that provide chemically reactive sites on the material surface can subsequently be grafted with various bioactive molecules, such as amino acids, proteins, other synthetic polymers, or nanoparticles. The grafted substances can further enhance the attractiveness of the modified polymer surface for cell adhesion and growth [18, 20–22]. 

In this study, we have therefore evaluated the adhesion, growth, and phenotypic maturation of vascular smooth muscle cells (VSMC) derived from rat aorta and cultured on low-density polyethylene (LDPE) modified by an Ar^+^ plasma discharge and subsequent grafting with glycine (Gly), bovine serum albumin (BSA), polyethylene glycol (PEG), colloidal carbon nanoparticles (C), or BSA+C. The aim of these modifications was to create surfaces attractive for cell colonization, and furthermore to be able to control the extent of cell adhesion, cell growth activity, and cell differentiation. Growth supports of this kind are particularly important for VSMC, in order to prevent their so-called phenotypic modulation, that is, transition from their quiescent differentiated state to a synthetic and proliferative phenotype (for a review, see [23]). This modulation is associated with the risk of restenosis of an artificial vascular graft. For this reason, VSMCs have been avoided in the construction of vascular replacements. However, VSMCs are the most numerous cell component of the natural blood vessel wall. Thus, these cells have to be included in advanced bioartificial vascular replacements, provided their phenotype, and proliferation activity is controlled by appropriate cell culture conditions, including the physical and chemical properties of their material carrier. 

LDPE was chosen as a carrier for VSMC in this study. Unlike other polymers, namely, polytetrafluoroethylene (PTFE) and polyethylene terephthalate (PET), LDPE is not used for constructing clinically applied vascular prostheses. However, due to its relatively simple and well-defined chemical composition, polyethylene provides a good model for studying the correlations between physicochemical changes of the material surface and the cell behavior. This model has been used with success in numerous earlier studies that we have carried out (e.g., [18, 20–22]). 

Another aim of this study was to compare the sensitivity of LDPE and high-density polyethylene (HDPE), used in most of our earlier studies, to plasma treatment and subsequent grafting, and the influence of these modifications on cell behavior. 

## 2. Experimental

### 2.1. Preparation of the Polymer Samples

The experiments were carried out on low-density polyethylene foils (LDPE) of Granoten S*H type (thickness 0.04 mm, density 0.922 g·cm^−3^, and melt flow index 0.8 g/10 minutes), made by the Granitol Joint-Stock Company, Moravsky Beroun, Czech Republic. The polyethylene samples were cut into circles (diameter 2 cm) using a metallic perforator. The foils were modified by an Ar^+^ plasma discharge (gas purity: 99.997%) using a Balzers SCD 050 device. The time of modification was 50 seconds, and the discharge power was 1.7 W. The chamber parameters were: Ar flow 0.3 l s^−1^, Ar pressure 10 Pa, electrode area 48 cm^2^, interelectrode electrode distance 50 mm, chamber volume 1000 cm^3^, and plasma volume 240 cm^3^. After this process, the samples were immersed in water solutions of glycine (Gly; Merck, Darmstadt, Germany, product number 104201), bovine serum albumin (BSA; Sigma-Aldrich, Germany, product number A9418) or polyethylene glycol (PEG; Merck, Darmstadt, Germany, product number 817018, m.w. 20000). Some plasma-treated samples and samples grafted with BSA were exposed to a suspension of colloidal carbon particles (C; Spezial Schwartz 4, Degussa AG, Germany) [18, 24]. All substances mentioned here were used in a concentration of 2 wt.%, and the time of the grafting process was 12 hours at room temperature [18]. Unmodified LDPE and standard cell culture polystyrene dishes were used as reference samples. 

### 2.2. Characterization of the Polymer Samples

It is known that after plasma treatment changes occur in a wide range of parameters in the modified layer during the aging process [25]. For this reason, all analyses (and also cell culture experiments) were performed 20 days after modification (i.e., on aged samples). 

The surface wettability was determined by measuring the contact angle using the static water drop method. The measurement was performed by distilled water in 10 different positions at room temperature with error 5% on the Surface Energy Evaluation System (SEE System, Advex Instruments, Czech Republic). 

The surface free energy (SFE) was evaluated on the basis of the contact angle of two liquids (water and glycerol). From these values, the SEE System calculates the surface energy according to the Owens-Wendt model [26].

The concentration profile of oxygen in the modified surface was determined by Rutherford Backscattering Spectroscopy (RBS). The RBS analysis was performed in a vacuum target chamber with 2.72 MeV He^+^ ions. The elemental depth profiles were inspected at an accessible depth of a few hundred nm. The RBS spectra were evaluated by the GISA 3.99 code. The typical RBS detection limit is 0.1 at.% for oxygen. 

The changes in surface morphology and roughness were determined by Atomic Force Microscopy (AFM), using a VEECO CP II in tapping mode. We used an RTESPA-CP Si probe, with spring constant 20–80 N/m. By repeated measurements of the same region (1 × 1 *μ*m^2^), it was proven that the surface morphology did not change after three consecutive scans. The mean roughness value (*R*_*a*_) is the arithmetic average of the deviations from the centre plane of the sample.

### 2.3. Cells and Culture Conditions

Each LDPE sample 2 cm in diameter was cut into four smaller parts of equal size, and these parts were used for evaluating the cell number and the cell morphology and for immunofluorescence staining. Whole samples were used for an evaluation of the molecular markers of cell adhesion and differentiation by enzyme-linked immunofluorescence assay (ELISA). All LDPE samples were sterilized with 70% ethanol for one hour. The smaller samples were inserted into 24-well plates (TPP, Switzerland; well diameter 1.5 cm), while for the bigger (i.e., whole) samples, 12-well plates from the same company (diameter 2.1 cm) were used. After sterilization, the samples were air-dried for 12 hours in a sterile environment in order to prevent possible negative effects of ethanol on the cells. After the drying process, the samples were fixed to the bottom of the culture wells by plastic rings (inner area 0.38 cm^2^ for the smaller samples and 1.77 cm^2^ for the bigger samples) in order to prevent the samples floating in the cell culture media. The samples were seeded with VSMC isolated by an explantation method from rat aorta. The cells were used in the third passage, and their seeding density was 17000 cells per cm^2^ [18]. The cells were cultivated in Dulbecco's Modified Eagle Minimum Essential Medium (DMEM, Sigma, USA), supplemented with 10% foetal bovine serum (Sebak GmbH, Aidenbach, Germany) and gentamicin (40 *μ*g/mL, LEK, Slovenia), for 1, 2, 4, 5, or 7 days (temperature 37°C, humidified atmosphere of 5% of CO_2_ in the air). For the smaller samples, 1.5 mL of the medium was used, and for the bigger samples, 3 mL of the medium. For the evaluation of the cell numbers and the cell spreading area, 4 smaller samples for each experimental group and time interval were used. For ELISA, 8 large samples for each experimental group were used, and the cultivation time was 7 days. For immunofluorescence staining, the time of cultivation was 4 days, and the cells were grown on the smaller samples.

### 2.4. Evaluation of the Cell Number and Cell Morphology

 The cells on the smaller polymer samples were rinsed in phosphate-buffered saline (PBS). On one sample per each experimental group, the cells were fixed by 70% cold ethanol (−20°C) and stained with a combination of Texas Red C_2_-maleimide fluorescent membrane dye (Molecular Probes, Invitrogen, Cat. number T6008; concentration 20 ng/mL in PBS) and Hoechst nuclear dye number 33342 (Sigma, USA; 5 *μ*g/mL in PBS). The cell number and cell morphology were then evaluated using an Olympus IX 51 microscope equipped with an Olympus DP 70 digital camera. 

The size of the cell spreading area was measured using Atlas software (Tescan Ltd, Czech Republic) on pictures taken on days 1 and 2 after seeding. 

On the three remaining smaller samples, the cells were counted in a Cell Viability Analyzer (Vi-cell XR, Beckman Coulter). After rinsing with PBS, the cells were harvested by 5 min treatment in a trypsin-EDTA solution (Sigma, Cat. number T4174). The device performed an automatic analysis of the number of viable and dead cells, based on the trypan blue exclusion test. 

### 2.5. Construction of Growth Curves and Calculation of Cell Population Doubling Time

The cell numbers obtained on days 1, 2, 5, and 7 after seeding were expressed as cells per cm^2^ (i.e., the cell population densities) and were used for constructing growth curves. The cell population doubling time (DT) was determined by the equation

(1) $$\begin{array}{c} \text{DT}=\log\left(2\right)\frac{t-t_{0}}{\log N_{t}-\log N_{t_{0}}}, \end{array}$$

where *N*_*t*_0__ and *N*_*t*_ are the cell population densities at the beginning and at the end of the studied culture interval [2, 3].

### 2.6. Immunofluorescence Staining

On day 4 after seeding, the cells were rinsed twice in PSB, fixed with precooled 70% ethanol (−20°C, 15 min), and pretreated with 1% bovine serum albumin in PBS containing 0.05% Triton X-100 (Sigma) for 20 minutes at room temperature, in order to block nonspecific binding sites and permeabilize the cell membrane. The cells were then incubated with primary antibodies against several molecular markers of adhesion and phenotypic maturation of VSMC, namely, integrin-associated focal adhesion proteins talin vinculin and paxillin, *α*-actinin, a protein present in focal adhesions and also binding the actin cytoskeleton, cytoskeletal protein *β*-actin, and contractile proteins *α*-actin and SM1 and SM2 myosins, markers of VSMC phenotypic maturation (Table 1). 

The primary antibodies were diluted in PBS to concentrations of 1 : 200 and applied overnight at 4°C. After rinsing with PBS, the secondary antibodies (dilution 1 : 400) were added for 1 hour at room temperature. These antibodies were goat anti-mouse or goat anti-rabbit F(ab′)2 fragments of IgG (H + L), both conjugated with Alexa Fluor 488 and purchased from Molecular Probes, Invitrogen (Cat. number A11017 and A11070, resp.). The cells were then rinsed twice in PBS, mounted under microscopic glass coverslips in a Gel/Mount permanent fluorescence-preserving aqueous mounting medium (Biomeda Corporation, Foster City, CA, U.S.A.), and evaluated under an Olympus IX 51 microscope (obj. 100x), using an Olympus DP 70 digital camera [18]. 

### 2.7. Enzyme-Linked Immunosorbent Assay (ELISA)

The differences in the concentration of the adhesion and differentiation molecules on the tested materials were evaluated semiquantitatively, using enzyme-linked immunosorbent assay (ELISA) in homogenized cells per mg of protein. 

For this purpose, the cells were cultured for 7 days and then rinsed with PBS, released with trypsin-EDTA solution (Sigma, Cat. number T4174, incubation 5 min at 37°C), and counted in the Cell Viability Analyzer (Vi-CELL XR, Beckman Coulter). Trypsinized cells were resuspended in PBS, centrifuged, resuspended in distilled and deionized water (10^6^ cells/in 200 *μ*L), and kept in a freezer at −70°C overnight. The cells were then homogenized by ultrasonication for 40 seconds (cycle 1, amplitude 70%) in a sonicator (UP 100 H, Dr. Hielscher GmbH), and the total protein content was measured using a modified method originally developed by Lowry [18, 27]. Aliquots of the cell homogenates corresponding to 1–50 *μ*g of protein in 50 *μ*L of water were adsorbed on 96-well microtiter plates (Maxisorp, Nunc) at 4°C overnight. After washing twice with PBS (100 *μ*L/well), the nonspecific binding sites were blocked by 0.02% gelatin in PBS (100 *μ*L/well, 60 min) and then treated by 1% Tween (Sigma, Cat. number P1379, 100 *μ*L/well, 20 min). The primary antibodies (Table 1) were diluted in PBS (1 : 200 to 1 : 500) and applied for 60 minutes at room temperature (50 *μ*L/well). The secondary antibodies, that is, goat anti-mouse IgG Fab specific (dilution 1 : 1000) or goat anti-rabbit IgG whole molecule (dilution 1 : 1000), both conjugated with peroxidase and purchased from Sigma (Cat. number A3682 and A0545, resp.), were applied for 45 min at room temperature (50 *μ*L/well). This step was followed by double washing in PBS and an orthophenylenediamine reaction (Sigma, Cat. number P1526, concentration 2.76 mM) using 0.05% H_2_O_2_ in 0.1 M phosphate buffer (pH 6.0, dark place, 100 *μ*L/well). The reaction was stopped after 10–30 minutes by 2 M H_2_SO_4_ (50 *μ*L/well), and the absorbance was measured at 490 and 690 nm by a Versa Max Microplate Reader (Molecular Devices Corporation, Sunnyvale, CA, USA). The absorbance of the cell samples taken from modified LDPE foils was given as a percentage of the value obtained in the cells on pure LDPE [18]. 

### 2.8. Statistics

The biological results were presented as mean ± SEM (Standard Error of Mean). The statistical significance was evaluated by the One-Way Analysis of Variance (ANOVA), Student-Newman-Keuls method. For the cell number and the size of the cell spreading area, multiple comparisons of values obtained on all tested samples were performed. For the data obtained from ELISA, values on tested samples were compared only with values on the control unmodified LDPE. For ELISA, Dunnett's posttest was also used. Values *P* ≤ 0.05 were considered significant.

## 3. Results and Discussion

### 3.1. Physicochemical Properties of Polymer Samples

**Figure 1:**
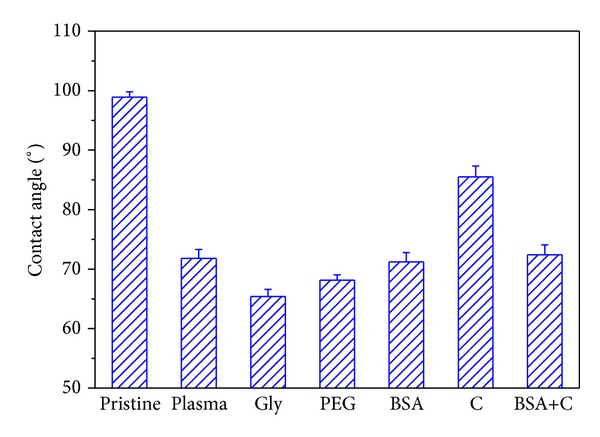
The water drop contact angle of pristine LDPE (pristine), plasma-treated LDPE (plasma), LDPE treated with plasma and then grafted with glycine (Gly), polyethylene glycol (PEG), bovine serum albumin (BSA), colloidal carbon particles (C), or a combination of BSA and C (BSA+C).

It is known that Ar plasma discharge can cause changes in the chemical structure and surface morphology of a modified layer. These changes significantly affect the surface wettability [28]. The degree of wettability, characterized by the contact angle, is shown in Figure 1 for pristine LDPE, plasma-treated LDPE, and plasma treated and subsequently grafted with biomolecules. The contact angle decreases dramatically after plasma treatment. This is due to the creation of free radicals and subsequent oxidation of the layer exposed to the air [29]. This leads to the formation of new oxygen structures, for example, carbonyl, carboxyl, and ester groups [30]. Subsequent grafting of glycine and PEG to the plasma-activated surface resulted in a further decrease in the contact angles on these samples. The molecules of Gly and PEG contain polar groups, which increase the hydrophilicity of the material surface. However, the presence of BSA and BSA+C had no significant effect on the wettability of LDPE, because the contact angles on these samples were comparable with the values on samples treated only with plasma. Different behavior was observed in samples after modification with colloidal C particles. These samples had much lower wettability than the plasma-treated samples. This result is in accordance with the previous finding by ourselves and by other authors that carbon-based coatings, for example, with amorphous diamond-like carbon, graphite or nonfunctionalized fullerenes, are often hydrophobic (for a review, see [19]). Nevertheless, the wettability of C-modified LDPE in this study still remained higher than on pristine LDPE.

**Figure 2:**
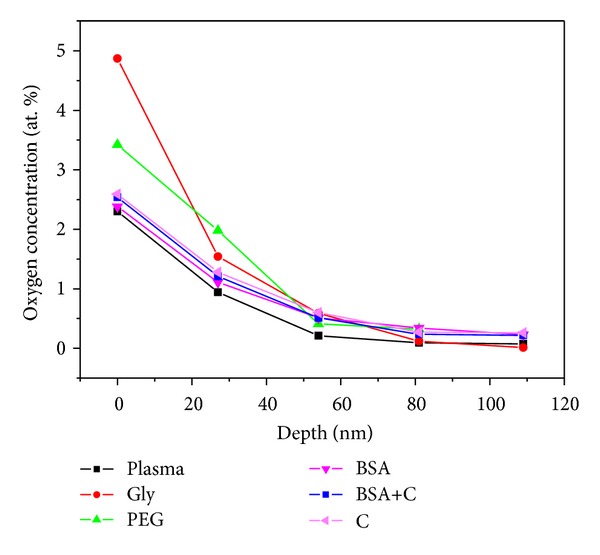
Oxygen concentration in the modified layer and depth concentration profile of oxygen, determined from RBS measurements, on plasma-treated LDPE (plasma), LDPE treated with plasma and then grafted with glycine (Gly), polyethylene glycol (PEG), bovine serum albumin (BSA), colloidal carbon particles (C), or a combination of BSA and C (BSA+C).

The total surface free energy (SFE) of the LDPE samples was inversely correlated with the water contact angle; that is, samples with a relatively low contact angle had a higher total SFE (LDPE grafted with Gly or PEG), while samples with a high contact angle had lower total SFE (pristine LDPE, Table 2). In other words, the total SPE was positively correlated with the hydrophilicity of the material surface. On all modified samples, the total SFE and its polar component increased in comparison with pristine LDPE. Similar differences between pristine and UV light-treated LDPE were described by O'Connell et al. [31]. The highest polar component of SFE was found on samples modified with plasma and subsequently grafted with PEG. This result is consistent with relatively high hydrophilicity of this sample (Figure 1), and also with the results obtained by RBS, which revealed a high concentration of oxygen in PEG-grafted LDPE (Figure 2). Surprisingly, the increase in the polar component was relatively small in LDPE grafted with Gly (Table 2), which exhibited the lowest water drop contact angle and the highest oxygen concentration at the material surface (Figures 1 and 2). In addition, the changes in the nonpolar component were variable. This component increased on samples modified in plasma or on samples subsequently grafted with glycin, while on samples grafted with BSA, C, or BSA+C, this value decreased. Similar variability has been described on LDPE, UHMWPE, and Sarlink polymers after treatment with UV light and has been explained by configuration changes of chemical functional groups at the material surface during its modification [31].

The concentration depth profile of oxygen modified LDPE was determined from RBS measurements. The concentration of oxygen in a modified layer and the oxygen depth profile is shown in Figure 2. The highest concentration of oxygen was found in the surface layers of all modified substrates. With increasing depth from the surface, the oxygen concentration decreased, and at a depth of 80 nm the amount of oxygen was minimal. Most oxygen in the modified layer was determined on a sample treated by plasma and grafted with glycine. More oxygen was also detected on a sample modified with plasma discharge and PEG. A comparable amount of oxygen was measured on substrates exposed to plasma or exposed to plasma and subsequently grafted with BSA or BSA+C. These results, obtained by the RBS method, are consistent with the results determined by goniometry, except that the samples grafted with C showed a similar oxygen content as the samples grafted with BSA and BSA+C, although their water drop contact angle was higher.

**Figure 3:**
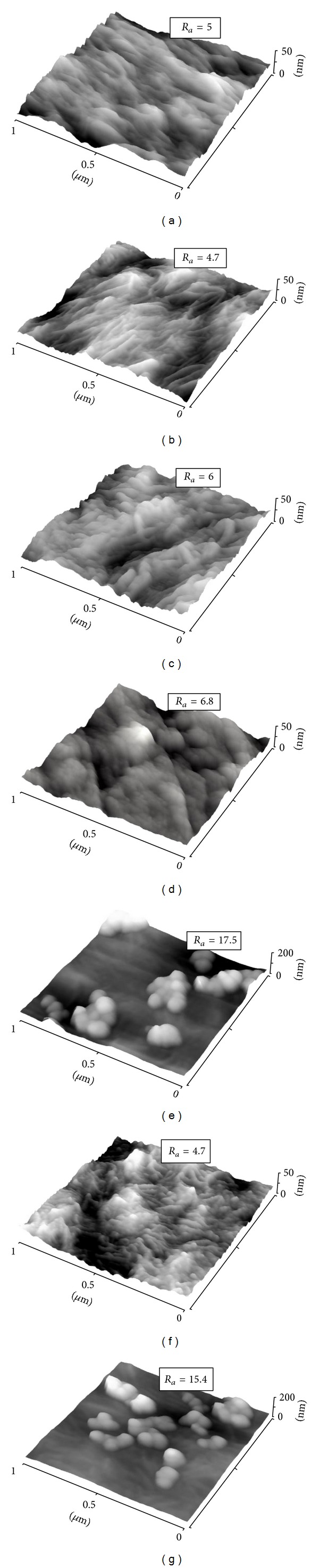
AFM scans of pristine LDPE (a), plasma-treated LDPE (b), LDPE treated with plasma and grafted with glycine (c), PEG (d), BSA (e), C (f), or BSA+C (g). *R*_*a*_ is the surface roughness in nm.

Changes in surface morphology and surface roughness that occurred due to plasma exposure and the subsequent grafting process were studied by the AFM method. AFM scans of pristine and modified samples are shown in Figure 3. Plasma modification leads to ablation of the surface layer. Its amorphous phase is ablated preferably [32]. This leads to enhanced branching of LDPE structures. Subsequent grafting with glycine and PEG on the plasma modified surface does not significantly change the surface morphology, but it slightly increases the surface roughness. Figure 3 shows that the bonding of carbon particles highlights the fine details of the structure and also leads to the formation of small spherical units corresponding to carbon nanoparticles. In the case of BSA and BSA+C grafting, large cluster formations that strongly affect the surface roughness were detected, in agreement with previous observations for BSA published by Wang et al. [33]. 

### 3.2. Initial Adhesion and Subsequent Growth of Cells on Polymer Samples

**Figure 4:**
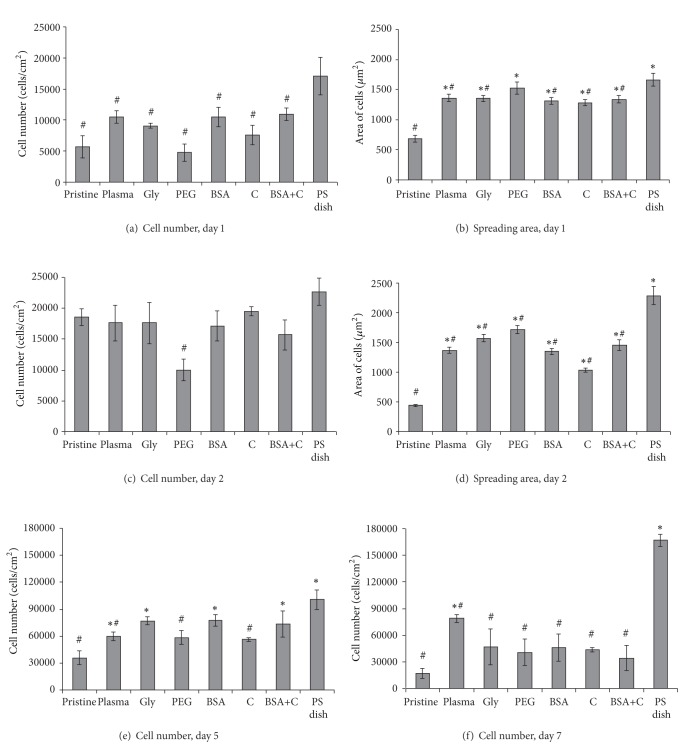
The number (a, c, e, and f) and the size of the spreading area (b, d) of rat aortic smooth muscle cells in cultures on nonmodified LDPE (pristine), on LDPE irradiated with plasma (plasma), on LDPE irradiated with plasma and grafted with glycine (Gly), on polyethylene glycol (PEG), on bovine serum albumin (BSA), on colloidal carbon particles (C), or on bovine serum albumin and C (BSA+C). A tissue culture polystyrene dish (PS dish) was used as a reference material. Days 1, 2, 5, and 7 after seeding. Mean ± SEM from 3 samples, each measured 50 times (cell number, Vi-CELL Analyser) or from 119 to 229 cells for each experimental group (spreading area). ANOVA, Student-Newman-Keuls method. Statistical significance: ^∗,#^ *P* ≤ 0.05 compared to the value on pristine PE and a polystyrene dish, respectively.

On the first day of the experiment, the highest number of initially adhered cells was found on the control PS dishes (17,100 ± 3,300 cells/cm^2^). The cell numbers on all LDPE samples showed no statistically significant differences, when compared with each other (Figure 4(a)). Nevertheless, the highest average cell number was observed on LDPE grafted with BSA (10,950 ± 1,030 cells/cm^2^), while the lowest number was found on LDPE grafted with PEG (4,770 ± 1,400 cells/cm^2^). This number was even slightly lower than the value found on pristine LDPE (5,720 ± 1,780 cells/cm^2^). This result contrasts with our earlier findings on HDPE foils [18], where the lowest initial cell number was found on the control PS, and the highest values were found on HDPE grafted with PEG. This disproportion in the cell behavior on LDPE and on HDPE could be explained by the different arrangements of the PEG chains on the surfaces of LDPE and HDPE samples. It is known that if PEG chains are attached to a surface only by one end, and dangle on the surface in a water environment (including cell culture media), this has a repulsive effect on cell adhesion. Cell adhesion on artificial materials *in vitro* is mediated by specific proteins adsorbed on the material surface from the serum supplement of the culture medium, mainly fibronectin and vitronectin. This adsorption is hampered or even disabled on mobile surfaces, especially if the surface mobility is combined with relatively high hydrophilicity, which occurs in PEG due to the presence of -OH groups in its molecules and which was also proven in this study (Figure 1). PEG—also known as polyethylene oxide (PEO), if its molecular mass is above 20,000—has been widely used for constructing surfaces that are nonadhesive for cells, especially those which were designed for the attachment of oligopeptidic ligands for cell adhesion receptors in defined types, concentrations, and distributions and which should prevent uncontrolled protein adsorption and aberrant cell adhesion [4, 34]; for a review, see [11–13]. PEG can also be attached to the material surface through several sites on one chain. In this case, PEG chains are not mobile and can support the adsorption of cell adhesion-mediating proteins and subsequent cell adhesion and growth [21]; for a review, see [18]. In our experiments, the cell-repulsive effects of PEG probably prevailed on LDPE, while PEG promoted cell adhesion on HDPE. This topic needs further investigation, but it can be supposed that the different behavior of PEG was due to the different structure of LDPE and HDPE. For example, LDPE molecules have more branching than HDPE molecules, so their intermolecular forces are weaker. The tensile strength of LDPE is lower, but its resilience is higher. Because of the side branches, LDPE molecules are less tightly packed and less crystalline than HDPE molecules. All these factors may lead to different attachment, distribution, and mobility of PEG chains on LDPE and HDPE surfaces, particularly if the branched LDPE structure is further enhanced by plasma treatment, as mentioned previously.

**Figure 5:**
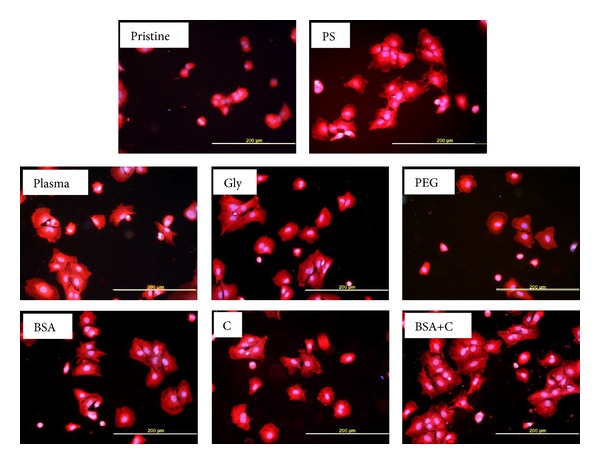
Morphology of rat aortic smooth muscle cells on day 1 after seeding on pristine LDPE (pristine), on a tissue culture polystyrene dish (PS), on LDPE irradiated with Ar^+^ plasma (plasma), on LDPE irradiated with plasma and grafted with glycine (Gly), on polyethyleneglycol (PEG), on bovine serum albumin (BSA), on colloidal carbon particles (C), or on bovine serum albumin and carbon particles (BSA+C). Cell membrane and cytoplasm stained with Texas Red C_2_-maleimide (red fluorescence), cell nuclei with Hoechst 33342 (blue fluorescence). Olympus IX 51 microscope, DP 70 digital camera, obj. 20x, bar = 200 *μ*m.

In spite of the relatively low number of initially adhered cells on PEG-grafted LDPE, the spreading area of cells on day 1 after seeding on this material (1,527 ± 98 *μ*m^2^) was on an average larger than the areas on the other modified LDPE samples (which ranged from 1,282 ± 49 *μ*m^2^ to 1,361 ± 60 *μ*m^2^), significantly larger than the areas on unmodified LDPE (687 ± 57 *μ*m^2^) and similar to those found on PS (1,664 ± 104 *μ*m^2^; Figure 4(b)). One explanation is that the cells had more space for spreading due to the lower number of adhering cells. In any case, the cell spreading implies that PEG-grafted LDPE surfaces allowed adsorption of cell adhesion molecules. However, this adsorption may be nonhomogeneous. On some regions, the cells were well spread and polygonal, while on other sites, the cells remained round (Figure 5). 

From day 1 to 2 after seeding, the cells on all LDPE-based samples started to enter the exponential phase of growth and proliferated with doubling times ranging on an average from 14.1 to 46.1 hours (Table 3), while the cells on polystyrene dishes remained rather in the lag phase and proliferated more slowly (doubling time 59.0 hours). As a result, the statistically significant differences between the LDPE samples and PS, observed on day 1, disappeared on day 2 after seeding (Figure 4(c)). Only on LDPE grafted with PEG did the cell number still remain significantly lower than on PS (10,000 ± 1,800 cells/cm^2^ versus 22,700 ± 2,300 cells/cm^2^ on PS). 

The cell spreading area on day 2 after seeding was (similarly as on day 1) significantly larger on all modified LDPE samples (1,037 ± 36 *μ*m^2^–1,714 ± 71 *μ*m^2^) than on unmodified LDPE (441 ± 18 *μ*m^2^), but at the same time it was significantly smaller than on PS (2,289 ± 149 *μ*m^2^; Figure 4(d)). Similar results were obtained in our earlier study performed on cultures on HDPE. The cell spreading areas of VSMC on HDPE treated with the same modifications as LDPE in the present study were larger than the spreading areas of the unmodified polymer. This can be explained by the increase in oxygen concentration on the surface of the modified polymer samples, their optimized wettability, and also changes in their surface roughness and morphology (Figures 1–3; [18]). 

**Figure 6:**
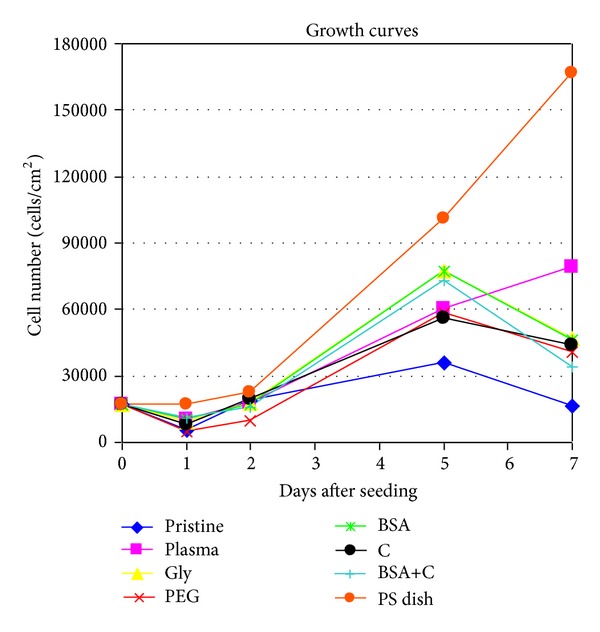
Growth dynamics of rat aortic smooth muscle cells in cultures on nonmodified PE (pristine), on PE irradiated with plasma (plasma), on PE irradiated with plasma and grafted with glycine (Gly), on polyethyleneglycol (PEG), on bovine serum albumin (BSA), on colloidal carbon particles (C), or on bovine serum albumin and C (BSA+C). A tissue culture polystyrene dish (PS dish) was used as the reference material. Means from three samples for each experimental group and time interval (each sample measured 50 times).

**Figure 7:**
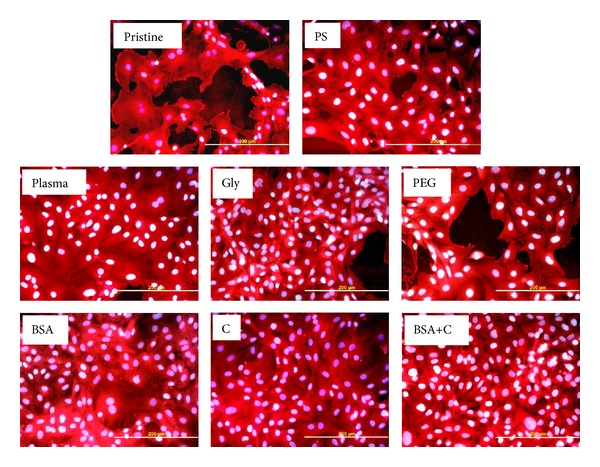
Morphology of rat aortic smooth muscle cells on day 5 after seeding on pristine LDPE (pristine), on a tissue culture polystyrene dish (PS), on LDPE irradiated with Ar^+^ plasma (plasma), on LDPE irradiated with plasma and grafted with glycine (Gly), on polyethyleneglycol (PEG), on bovine serum albumin (BSA), on colloidal carbon particles (C), or on bovine serum albumin and carbon particles (BSA+C). Cell membrane and cytoplasm stained with Texas Red C_2_-maleimide (red fluorescence), cell nuclei with Hoechst mumber 33342 (blue to white fluorescence). Olympus IX 51 microscope, DP 70 digital camera, obj. 20x, bar = 200 *μ*m.

It is known that the size of the spreading area correlates positively with cell proliferation activity, at least to a certain extent [35, 36]; for a review, see [11–13]. In accordance with this, from day 2 to 5 after seeding, the cells on all modified samples proliferated more quickly (doubling time from 9.4 to 13.6 hours) than the cells on pristine LDPE (doubling time 25.5 hours); see Table 3, Figure 6. On day 5 after seeding, the cells on the modified LDPE samples (except the samples grafted with PEG and C) reached a significantly higher cell population density (59,940 ± 4,850–77,530 ± 6,460 cells/cm^2^) than the cells on pristine LDPE (35,680 ± 7,760 cells/cm^2^). On LDPE grafted with Gly, BSA, or BSA+C, the cell population densities reached values not significantly different from those obtained on the PS dishes (100,740 ± 10,930 cells/cm^2^), which are considered as the “gold standard” for cell cultivation (Figure 4(e)). The samples grafted with BSA or BSA+C were covered by almost confluent cell layers (Figure 7). Similar results were also obtained in our earlier study performed on HDPE, but the cell population densities on BSA and BSA+C significantly exceeded the values on the PS dishes [18].

The improved spreading and growth of VSMC on LDPE irradiated with plasma can be explained by physicochemical changes in the polymer surface which lead to an increased oxygen content and increased wettability of the surface. These surfaces are then more susceptible to adsorption of adhesion-mediating extracellular matrix proteins, for example, vitronectin, fibronectin, collagen, or laminin, in a near physiological and bioactive conformations, suitable for binding by the cell adhesion receptors (for a review, see [11–13]). 

The reactive sites formed in a polymer when it is exposed to plasma can be used for grafting various molecules which further modulate the effects of polymer treatment on cell adhesion, growth, phenotypic maturation, and functioning. Glycine, used for grafting the plasma-activated LDPE in this study, is a component of a well-known ligand for integrin cell adhesion receptors, that is, Arg-Gly-Asp (RGD), though Gly alone cannot be bound by these receptors. Nevertheless, Gly grafting enriches the irradiated polymer surface with additional oxygen-containing and also amine groups, which are known to improve the adsorption of cell adhesion-mediating proteins and cell colonization [16]; for a review, see [12]. In addition, Gly and PEG slightly increased the size of the nanoscale irregularities on the polymer surface; that is, they enhanced the material nanostructure, which has been shown, like surface wettability, to optimize the spectrum and the geometrical conformations of adsorbed cell adhesion-mediating molecules (for a review, see [12]). 

Albumin, a protein that is nonadhesive for cells, has been used for constructing cell-repulsive surfaces. From this point of view, our result that albumin grafting improved the colonization of LDPE with cells might be surprising. However, albumin preadsorbed on the material surfaces improved the geometrical conformation of the cell adhesion-mediating molecules, for example, fibronectin and vitronectin, and their accessibility for cell adhesion receptors [37, 38]; for a review, see [12, 18]. In addition, BSA grafted on the LDPE formed nanosized (i.e., smaller than 100 nm) and submicron-sized (i.e., smaller than 1 *μ*m) cluster structures (Figure 3). Irregularities of this size are believed to promote the adsorption of cell adhesion-mediating molecules in a more physiological conformation than the conventional flat surfaces, and this markedly improves the adhesion and further functioning of the cells [39, 40].

 From day 5 to 7 after seeding, the cells on most modified LDPE samples stopped proliferating (i.e., entered the stationary phase of growth), and they even decreased in number. Cell proliferation continued only on the polystyrene dishes and on the plasma-irradiated LDPE (Figure 6, Table 3). On all LDPE samples, the final cell population density was significantly lower than on the PS dishes (Figure 4(f)). However, the cell proliferation on all modified HDPE samples in our earlier study continued from day 5 to 7, and the final cell population density on these samples was similar to or even higher than on the PS dishes [18]. 

Thus, the positive effects of plasma treatment and subsequent grafting on the adhesion and growth of VSMC were more apparent in HDPE than in LDPE. Analogous results were obtained when a terpolymer of polytetrafluoroethylene, polyvinyldifluoride and polypropylene (PTFE/PVDF/PP), and polysulfone (PSU) was mixed with carbon nanotubes. The addition of nanotubes significantly improved the adhesion and growth of human osteoblast-like MG 63 cells on the terpolymer, while this effect was much less apparent on PSU. This difference was explained by the high hydrophobicity of pristine PTFE/PVDF/PP (water drop contact angle ~100°), while the pristine PSU was more hydrophilic (contact angle ~85°). In addition, PSU contains bioactive sulphone groups, which can also mediate the positive effects of this polymer on cell adhesion. Sulphonated polystyrene promoted the adsorption of fibronectin in an advantageous geometrical conformation for cell adhesion [41]. Thus, pristine PSU is more suitable for cell colonization, and thus the cells were less sensitive to the modifications, further improving the cell adhesion and growth (for a review, see [42]). However, in our studies, both LDPE and HDPE were relatively hydrophobic, having a similar water drop contact angle of 98.6 ± 1.90° and 102.5 ± 2.31°, respectively. Thus, the different sensitivity of cells to modifications of LDPE and HDPE may be due to other differences in the physical and chemical properties of these polymers (see above), for example, differences in chain branching, tensile strength, resilience, crystallinity, packing density, and so forth. A further investigation of this topic is needed.

### 3.3. Distribution and Concentration of Molecular Markers of Cell Adhesion and Phenotypic Maturation

**Figure 8:**
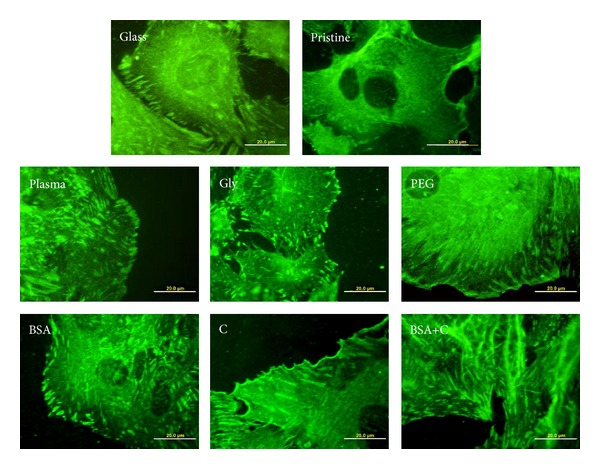
Immunofluorescence of talin, an integrin-associated protein of focal adhesion plaques, in rat aortic smooth muscle cells on day 4 after seeding on a microscopic glass coverslip (glass), on pristine LDPE (pristine), on LDPE irradiated with plasma (plasma), on LDPE irradiated with Ar^+^ plasma and grafted with glycine (Gly), on polyethylene glycol (PEG), on bovine serum albumin (BSA), on colloidal carbon particles (C), and on bovine serum albumin with C (BSA+C). Olympus IX 51 microscope, DP 70 digital camera, obj. 100x, bar = 20 *μ*m.

Immunofluorescence staining showed that the cells on all modified LDPE samples had better developed focal adhesion plaques containing talin and vinculin than the cells on pristine LDPE samples (Figure 8). This result correlates well with the size of the cell spreading area, which was generally larger on all modified samples than on pristine LDPE. 

**Figure 9:**
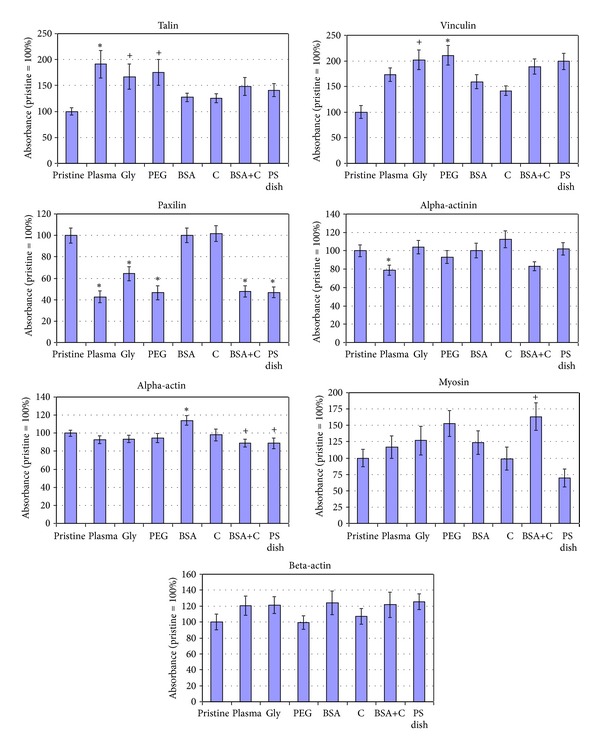
Concentration of focal adhesion, cytoskeletal, and contractile proteins in rat aortic smooth muscle cells in 7-day-old cultures on pristine PE (pristine), on PE activated with plasma (plasma), on PE grafted with glycine (Gly), on polyethylene glycol (PEG), on bovine serum albumin (BSA), on colloidal carbon particles (C), or on BSA with C (BSA+C), and on a tissue culture polystyrene dish (PS dish). Measured by ELISA per mg of protein. Means ± SEM from three to seven experiments, each performed in duplicate or in triplicate. Absorbance values were normalized to the values obtained in cell samples from pristine PE, that is, given as a percentage of the values on pristine PE. ANOVA, statistical significance: ^∗,+^ *P* ≤ 0.05 compared to the value on pristine PE (Student-Newman Keuls method and Dunnett's posttest, resp.).

ELISA revealed that the concentration of talin in cells on LDPE modified with plasma, or plasma with subsequent grafting of Gly or PEG, was significantly higher than in cells on pristine LDPE. The concentration of vinculin, which stabilizes focal adhesion plaques (for a review, see [12]), was also higher in cells on LDPE grafted with Gly or PEG (Figure 9). In accordance with these findings, the cells on LDPE modified with plasma, Gly, and particularly PEG achieved on an average the largest cell spreading areas (Figures 4(b) and 4(d)). 

**Figure 10:**
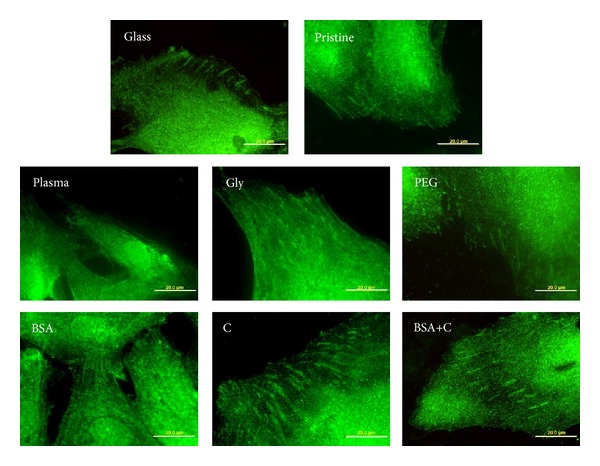
Immunofluorescence of paxillin, an integrin-associated protein of focal adhesion plaques, in rat aortic smooth muscle cells on day 4 after seeding on a microscopic glass coverslip (glass), on pristine LDPE (pristine), on LDPE irradiated with plasma (plasma), on LDPE irradiated with Ar^+^ plasma and grafted with glycine (Gly), on polyethylene glycol (PEG), on bovine serum albumin (BSA), on colloidal carbon particles (C), and on bovine serum albumin with C (BSA+C). Olympus IX 51 microscope, DP 70 digital camera, obj. 100x, bar = 20 *μ*m.

However, the concentration of paxillin (i.e., another protein of focal adhesion plaques) was lower in cells on LDPE modified with plasma, Gly, PEG, and BSA+C than in cells on pristine LDPE (Figure 9). Only in cells on LDPE modified with BSA or C the concentration of paxillin was similar as in the cells on pristine polymer. This result corresponded, at least partly, with immunofluorescence staining of paxillin (Figure 10). In cells on LDPE modified with plasma, Gly, or PEG, the paxillin-containing focal adhesions were equally developed or even worse developed than in cells on pristine LDPE, while the paxillin focal adhesions on polymer modified with BSA or C were similarly developed or better developed than in cells on pristine LDPE. Only in cells on BSA+C the paxillin-containing focal adhesions were well-developed, though the concentration of paxillin in these cells was relatively low (Figures 9 and 10). In this context, it should be pointed out that the ELISA method used in our study measured the total number of focal adhesion molecules in the cells, and not only the molecules located in focal adhesion plaques. A more exact method might be to extract the cytosolic fraction of the focal adhesion proteins (i.e., not bound in the focal adhesion plaques) by detergents [43] or to use antibodies against phosphorylated antigens. It can be supposed that phosphorylated paxillin (i.e., activated paxillin exerting its function in cell adhesion) is located in the focal adhesion plaques, while in the cytosolic fraction, nonactive paxillin is not phosphorylated [44–46].

**Figure 11:**
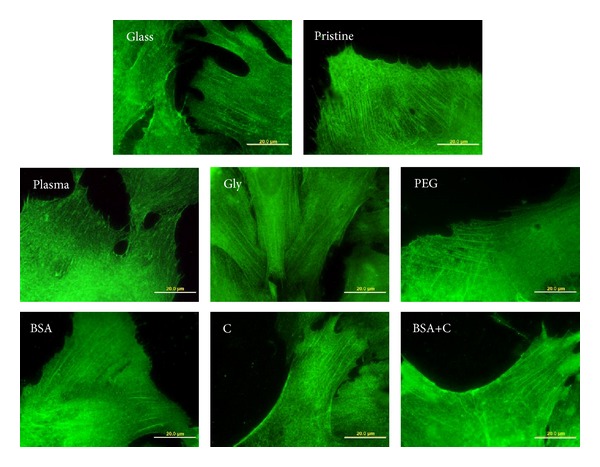
Immunofluorescence of *α*-actinin, a protein associated with both focal adhesion plaques and the actin cytoskeleton, in rat aortic smooth muscle cells on day 4 after seeding on a microscopic glass coverslip (glass), on pristine LDPE (pristine), on LDPE irradiated with plasma (plasma), on LDPE irradiated with Ar^+^ plasma and grafted with glycine (Gly), on polyethylene glycol (PEG), on bovine serum albumin (BSA), on colloidal carbon particles (C), and on bovine serum albumin with C (BSA+C). Olympus IX 51 microscope, DP 70 digital camera, obj. 100x, bar = 20 *μ*m.

Alpha-actinin is associated with both focal adhesion plaques and the actin cytoskeleton, in which it acts as a crosslinker and stabilizer of the contractile apparatus [47, 48]. In cells on the modified LDPE samples and on PS, its concentration was usually similar to that in cells on pristine LDPE (Figure 9). Only in cells on plasma-irradiated LDPE the concentration of *α*-actinin was significantly lower. From the cells on all modified samples, only the cells on plasma-modified LDPE continued their proliferation from day 5 to 7 (Figure 6, Table 3). Thus, the decreased concentration of *α*-actinin may be a sign of certain instability of the actin cytoskeleton, which is reorganized during cell division. This presumption is further supported by the immunofluorescence staining pattern of *α*-actinin. In cells on plasma-modified LDPE, the structures containing *α*-actinin are relatively short and are located predominantly at the cell periphery, while in the cells on the other samples, the *α*-actinin-containing structures are long, filament like, often running in parallel through the entire cell (Figure 11).

VSMCs exist in two basic phenotypes, referred to as contractile and synthetic. The contractile phenotype occurs in mature healthy blood vessels and is characterized by the presence of desmin, that is, a protein of intermediate filaments, muscle type of tropomyosin, T-troponin, h-caldesmon, h1-calponin, and metavinculin, and particularly by contractile proteins *α*-actin and SM1 and SM2 isoforms of myosin. On the other hand, the synthetic phenotype is characterized by the accumulation of cell organelles involved in proteosynthesis, such as endoplasmic reticulum, ribosomes or Golgi complex, and predominance of *β*- and *γ*-isoforms of actin and nonmuscle myosin. This phenotype occurs physiologically in immature blood vessels under development, pathologically in diseased blood vessels, for example, during atherosclerosis and hypertension, and artificially after disintegration of the vascular wall and seeding VSMC *in vitro* (for a review, see [48]). However, the synthetic phenotype of VSMC *in vitro* can be reversed, at least partly, to the contractile phenotype by appropriate cell culture conditions, such as the use of serum-free media, dynamic stimulation of VSMC, and particularly the physical and chemical properties of the adhesion substrate. In cells on our materials, the distribution and concentration of *α*-actin or SM1 and SM2 myosins were therefore evaluated as markers of phenotypic maturation of VSMC, that is, their transition toward a more differentiated contractile phenotype. 

**Figure 12:**
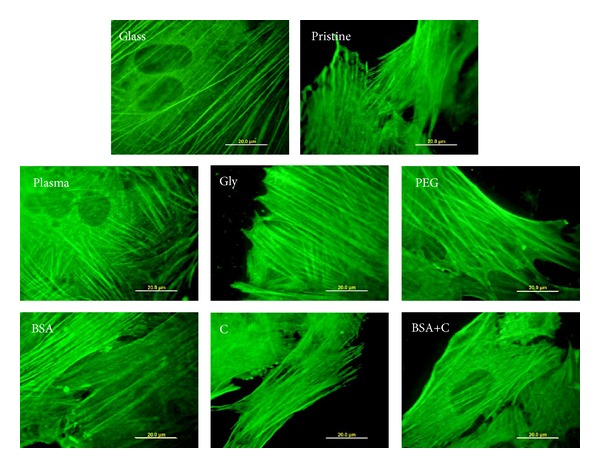
Immunofluorescence of contractile protein *α*-actin in rat aortic smooth muscle cells on day 4 after seeding on a microscopic glass coverslip (glass), on pristine LDPE (pristine), on LDPE irradiated with plasma (plasma), on LDPE irradiated with Ar^+^ plasma and grafted with glycine (Gly), on polyethylene glycol (PEG), on bovine serum albumin (BSA), on colloidal carbon particles (C), and on bovine serum albumin with C (BSA+C). Olympus IX 51 microscope, DP 70 digital camera, obj. 100x, bar = 20 *μ*m.

**Figure 13:**
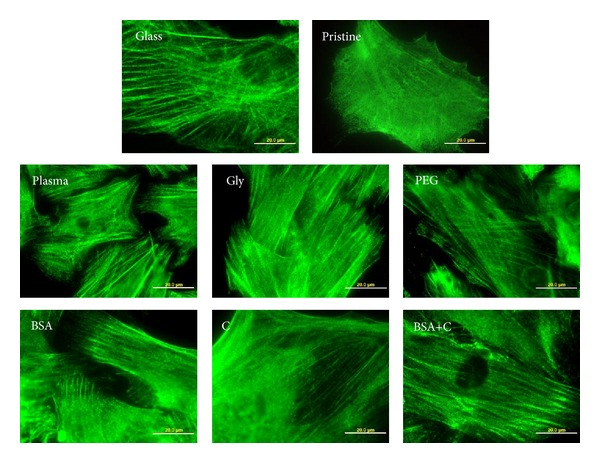
Immunofluorescence of contractile proteins SM1 and SM2 myosins in rat aortic smooth muscle cells on day 4 after seeding on a microscopic glass coverslip (glass), on pristine LDPE (Pristine), on LDPE irradiated with plasma (plasma), on LDPE irradiated with Ar^+^ plasma and grafted with glycine (Gly), on polyethylene glycol (PEG), on bovine serum albumin (BSA), on colloidal carbon particles (C), and on bovine serum albumin with C (BSA+C). Olympus IX 51 microscope, DP 70 digital camera, obj. 100x, bar = 20 *μ*m.

We found that *α*-actin formed thick and brightly stained filament bundles in cells on all samples, including pristine LDPE (Figure 12), while bundles containing SM1 and SM2 myosins were more apparent in cells on modified LDPE than on pristine LDPE (Figure 13). However, as revealed by ELISA, the concentration of *α*-actin and SM1 and SM2 myosins was significantly higher only in cells on LDPE grafted with BSA and BSA+C, respectively, compared to cells on pristine LDPE (Figure 9). The cells on the samples grafted with BSA or BSA+C had a relatively high cell population density on day 5 after seeding, approaching the value found on cell culture PS, and reached confluence. In addition, these cells had relatively large spreading areas and well-developed talin- and vinculin-containing focal adhesion plaques (Figures 4(b), 4(d), and 8). These factors, that is, good cell-cell adhesion in confluent cultures together with good cell-matrix adhesion, are associated with cell differentiation (for a review, see [11–13]). At the same time, the concentration of *β*-actin, that is, an actin isoform nonspecific for VSMC and also present in nonmuscle cell types, was similar in the cells on all tested samples (Figure 9). 

Taken together, like the changes in cell number and in spreading area, the changes in the concentration of molecular markers of the adhesion and maturation of VSMC were also generally less apparent on LDPE than on HDPE, after the same modifications of the two polymers (for comparison, see [18]). LDPE grafted with Gly or PEG supported mainly the cell spreading and formation of talin- and vinculin-containing focal adhesion plaques, while the functionalization of LDPE with BSA or BSA+C tended to promote the growth of VSMC to high population densities and their phenotypic maturation, manifested by a higher concentration of *α*-actin and SM1 and SM2 myosins.

## 4. Conclusion and Further Perspectives

Modifications of LDPE samples with an Ar^+^ plasma discharge and subsequent grafting with glycine, PEG, BSA, C particles, and BSA with C particles improved the colonization of LDPE with VSMC. Grafting with glycine or PEG mainly increased the cell spreading and the concentration of focal adhesion proteins talin and vinculin, which could be attributed to the relatively high oxygen content and wettability of these samples. Grafting LDPE with BSA and BSA+C supported the growth of VSMC to confluence and increased the concentration of *α*-actin and myosins (SM1 and SM2) in these cells. This could be explained by an enhanced nano- and submicron-scale structure and also by the wettability of the samples. Thus, the plasma treatment and grafting with bioactive substances used in this study improved the adhesion, growth, and phenotypic maturation of VSMC, though these changes were less pronounced than those observed on HDPE in our earlier study [18]. Nevertheless, the polymer modifications developed in this study have a potential for creating a bioartificial vascular wall with reconstructed *tunica media*. 

## Figures and Tables

**Table 1:** Primary antibodies used for immunofluorescence staining (Immf.) and enzyme-linked immunosorbent assay (ELISA) of markers of adhesion and phenotypic maturation of VSMCs.

Antibody against	Developed in, type	Company, Cat. number	Dilution	Incubation
Chicken talin	Mouse, monoclonal	Sigma^a^ T 3287	Immf.: 1 : 200 ELISA: 1 : 500	Immf.: overnight, 4°C ELISA: 60 min, RT^c^
Human vinculin	Mouse, monoclonal	Sigma^a^ V9131	Immf.: 1 : 200 ELISA: 1 : 400	Immf.: overnight, 4°C ELISA: 60 min, RT^c^
Recombinant human paxillin	Rabbit, polyclonal	Chemicon^b^ P1093	Immf.: 1 : 200 ELISA: 1 : 400	Immf.: overnight, 4°C ELISA: 60 min, RT^c^
Chicken *α*-actinin	Rabbit, polyclonal	Sigma^a^ A2543	Immf.: 1 : 200 ELISA: 1 : 500	Immf.: overnight, 4°C ELISA: 60 min, RT^c^
Synthetic peptide of *α*-smooth muscle actin	Mouse, monoclonal	Sigma^a^ A2547	Immf.: 1 : 200 ELISA: 1 : 400	Immf.: overnight, 4°C ELISA: 60 min, RT^c^
Synthetic peptide of *β*-actin	Mouse, monoclonal	Sigma^a^ A 5441	Immf.: 1 : 200 ELISA: 1 : 400	Immf.: overnight, 4°C ELISA: 60 min, RT^c^
Human smooth muscle SM1 and SM2 myosin	Mouse, monoclonal	Sigma^a^ M7786	Immf.: 1 : 200 ELISA: 1 : 500	Immf.: overnight, 4°C ELISA: 60 min, RT^c^

^a^Sigma, St. Louis, MO, USA; Czech Dealer: Sigma-Aldrich S.R.O., Prague, Czech Republic.

^b^Chemicon International Inc., Temecula, CA, USA; Czech dealer: Scintilla S.R.O., Jihlava, Czech Republic.

^c^Room temperature (RT).

**Table 2:** Surface free energy (SFE, in mJ·m^−2^) of pristine LDPE, plasma-treated LDPE (plasma), LDPE treated with plasma and then grafted with glycine (Gly), polyethylene glycol (PEG), bovine serum albumin (BSA), colloidal carbon particles (C), or with a combination of BSA and C (BSA+C). *γ*_total_ is the total SFE of the polymer and consists of nonpolar (*γ*_LW_) and polar components (*γ*_AB_).

	*γ*_total_	*γ*_LW_	*γ*_AB_
Pristine	24.54	23.16	1.37
Plasma	28.56	25.01	3.55
Gly	36.20	31.89	4.31
PEG	34.91	5.01	29.90
BSA	26.56	20.28	6.28
C	26.59	19.22	7.37
BSA+C	25.58	12.86	12.72

The values are the mean of 10 to 15 measurements for each experimental group and were calculated automatically by the Surface Energy Evaluating System (SEE System, Masaryk University, Brno, Czech Rep.).

**Table 3:** Cell population doubling time (hours) of rat aortic smooth muscle cells from day 1 to 2, day 2 to 5, and day 5 to 7 after seeding on nonmodified LDPE (pristine), LDPE irradiated with plasma (plasma), LDPE irradiated with plasma and grafted with glycine (Gly), polyethyleneglycol (PEG), bovine serum albumin (BSA), colloidal carbon particles (C), bovine serum albumin and C (BSA+C), and polystyrene dishes (PS dish).

Material/time interval	Pristine	Plasma	Gly	PEG	BSA	C	BSA+C	PS dish
Days 1-2	14.1	32.0	25.0	22.5	33.8	17.7	46.1	59.0
Days 2–5	25.5	13.6	11.3	9.4	11.0	15.7	10.8	11.2
Days 5–7	−21.8*	60.3	−33.6*	−45.5*	−32.1*	−66.9*	−21.9*	33.0

*Nonproliferating cells.
